# Active Self-Assembly of Ladder-Shaped DNA Carrier for Drug Delivery

**DOI:** 10.3390/molecules28020797

**Published:** 2023-01-13

**Authors:** Yuan Liu, Jiaxin Wang, Lijun Sun, Bin Wang, Qiang Zhang, Xiaokang Zhang, Ben Cao

**Affiliations:** 1 School of Computer Science and Technology, Dalian University of Technology, Dalian 116024, China; 2 Key Laboratory of Advanced Design and Intelligent Computing, Dalian University, Ministry of Education, Dalian 116622, China

**Keywords:** DNA nanotechnology, DNA self-assembly, DNA tiles, drug delivery, drug carrier

## Abstract

With the advent of nanotechnology, DNA molecules have been transformed from solely genetic information carriers to multifunctional materials, showing a tremendous potential for drug delivery and disease diagnosis. In drug delivery systems, DNA is used as a building material to construct drug carriers through a variety of DNA self-assembly methods, which can integrate multiple functions to complete in vivo and in situ tasks. In this study, ladder-shaped drug carriers are developed for drug delivery on the basis of a DNA nanoladder. We first demonstrate the overall structure of the nanoladder, in which a nick is added into each rung of the nanoladder to endow the nanoladder with the ability to incorporate a drug loading site. The structure is designed to counteract the decrement of stability caused by the nick and investigated in different conditions to gain insight into the properties of the nicked DNA nanoladders. As a proof of concept, we fix the biotin in every other nick as a loading site and assemble the protein (streptavidin) on the loading site to demonstrate the feasibility of the drug-carrying function. The protein can be fixed stably and can be extended to different biological and chemical drugs by altering the drug loading site. We believe this design approach will be a novel addition to the toolbox of DNA nanotechnology, and it will be useful for versatile applications such as in bioimaging, biosensing, and targeted therapy.

## 1. Introduction

DNA nanotechnology takes advantage of DNA structures and functions that have not yet been used in the natural world [1,2], which makes DNA not only function as a genetic information carrier [3,4,5], but also act as a role of programmable material [6,7]. Owing to the distinct advantages of programmability and predictability, DNA molecules have been used for fabricating two- or three-dimensional nanostructures by a precise sequence design [8,9]. Researchers hope to use DNA to assemble desired structures through an artificial construction, which can be used in biology [10,11], medicine [12,13], artificial intelligence [14], information storage [15,16], and other fields. Due to the distinct advantages of its biocompatibility, molecular recognition ability, and ease of preparation, DNA self-assemblies are excellent candidates to build drug carriers in drug delivery systems [17,18]. DNA self-assemblies typically exhibit a low cytotoxicity and high resistance to an enzymatic degradation in biological media [19]. DNA tetrahedrons can remain substantially intact for at least 48 h after a transfection into cells [20]. Aptamer-tethered DNA nanoassemblies showed little changes after being treated with a relatively high concentration of DNase I for 24 h, which is more than twice the concentration of DNase in human blood [21].

**Figure 1:**
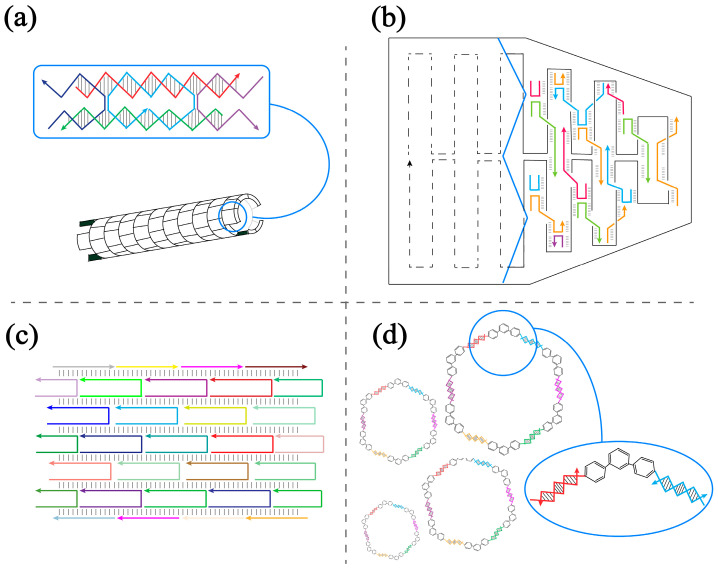
Scheme of self-assembly methods. The solid line with an arrow represents a single strand of DNA. (**a**) Tile-based self-assembly method. (**b**) DNA origami-based self-assembly method. (**c**) DNA brick-based self-assembly method. (**d**) Supramolecular DNA assembly method.

A variety of design rules and self-assembly methods have been developed to construct predictable and programmable DNA materials into increasingly complex nanostructures. Tile-based self-assembly methods [22,23] assemble basic motifs into structures through a periodic interaction between the motifs (Figure 1a). DNA origami-based self-assembly methods [24,25] utilize many short staple strands to bind a long, single DNA strand and fold it into a certain shape (Figure 1b). DNA brick-based self-assembly methods [26,27] combine hundreds of single strands into a designed structure, and the design process is similar to filling a canvas with pixels (Figure 1c). Supramolecular DNA assembly methods [7] use nucleic acids and non-nucleic acid materials to create novel nanostructures together, which can increase the diversity of DNA-based nanostructures and functions (Figure 1d). Among the aforementioned methods, tile-based self-assembly methods require only a few DNA strands to assemble a basic motif, which is then further grown into nanostructures. This reduces the design complexity and assembly error rate, making tile-based self-assembly methods the top choice of many researchers. The design of the tiles comes from the Holliday junction [28], which is a DNA branched junction appearing in genetic recombination. The branched junction can be immobilized by eliminating the two-fold symmetry at the junction [7]. Afterwards, by introducing crossovers, rigid motifs with a controllable connectivity can be designed, such as the DX tile [29] and N-point star [30,31]. These rigid motifs are widely used in the self-assembly of two-dimensional [9] and even three-dimensional [8] nanostructures. In order to further expand the application scope and enrich the types and functions of the tiles, Shogo Hamada and Satoshi Murata broke through the original design limitations. They combined several crossover junctions and proposed a new structure called a T-junction [32], a single duplex-based structure. The direction of the connection between two T-junction motifs can be changed by adjusting the length of the double and single strand. As a building block different from traditional structures, the T-junction ensures structural flexibility while providing rigidity, enabling the assembly of single duplex-based branched motifs into structures and providing more usable shapes for building nanostructures. Thus, the basic motif with a “Z” or “C” shaped structure can be designed and assembled into various types of nanostructures.

Moreover, inspired by a dynamic regulation in biological systems, the response trigger phenomenon has been applied to tile-based assembly. There are two design methods for the responsive assembly of tiles depending on whether the external input directly activates the tiles. For responsive assembly in which the external input does not directly activate the tiles, the assembly process is usually controlled by upstream circuits. The upstream circuits are activated by external inputs and then the assembly of downstream tiles can be initiated. Such inputs include nucleic acids [33], pH changes [34], light [35], and antibodies [36]. For a responsive assembly in which the external input directly activates the tiles, the assembly process usually relies on changes in the tile conformation, which requires the careful design of the basic motif to be allosteric in response to external inputs. Basic motifs can be designed using changes in the rigidity of the tile [23,37] or in combination with special structures; these are usually hairpin structures [38]. The catalytic hairpin assembly reaction (CHA) [39] and hybridization chain reaction (HCR) [40] are two reactions realized with hairpin structures that can be used to change the tile conformation for a responsive assembly. Among them, an HCR is often used for DNA self-assembly due to its isothermal condition, simple operation, and behavior that can generate long DNA double helices after being triggered.

A single HCR reaction can only form simple double helix structures, whereas more complex DNA nanostructures can be formed if multiple HCRs or different mechanisms [41] are combined. Furthermore, an HCR is a natural response switch due to its trigger-response mechanism. An HCR can be combined with T-junction cohesion to cause the existing T-junction to adjust its structure to develop more kinds of assembly, including responsive assembly. This brings new options for the application of responsive assembly in sensing and nanomachines.

In this study, we report a ladder-shaped DNA drug carrier, in which a hybridization chain reaction and the T-junction collaborate to connect the DNA tiles. In order to empower the nanoladder with the ability to carry a drug, we add a nick to each rung of the nanoladder to enable the structure to incorporate drug loading sites. To eliminate the impact of the remarkable reduction in the binding strength caused by the nick, we increase the length of the rungs to 21 bps. On the basis of the lengthened rungs, we redesigned the tiles so that they can self-assemble after being triggered. Then, we investigated the properties and stability of the nanoladders by conducting experiments on the incubation time, incubation temperature, and ratio of the initiator to tiles. After determining the best conditions, we explore them as a pathway to construct a drug carrier. The results show that the ladder-shaped drug carriers are rigid enough to load a 66 kDa tetrameric protein, i.e., streptavidin (STV), in every drug loading site. We believe that our drug carriers have the ability to carry many kinds of drugs that can meet the requirements for diagnosis and therapy, and can serve as important tools for the study of biosensors [42], structural scaffolds [43], molecule machines [44], and more.

## 2. Results and Discussion

### 2.1. Design of DNA Nanoladders

**Figure 2:**
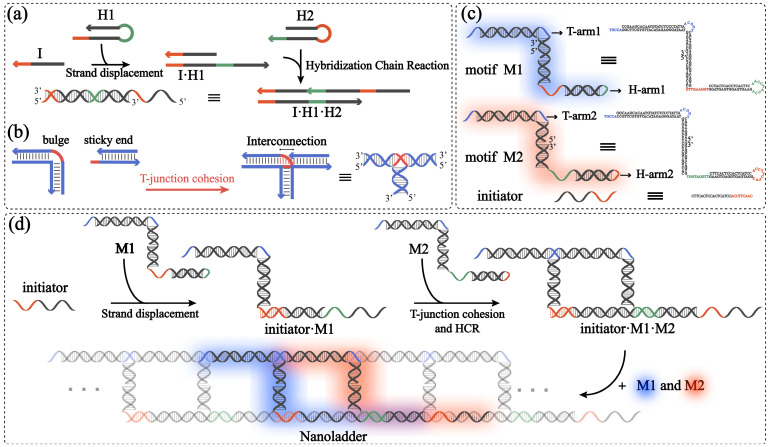
(**a**) Schematic representation of hybridization chain reaction (HCR). (**b**) Schematic representation of T-junction. (**c**) Schematic illustrations of structures (**left**), and sequences of motif M1, motif M2, and initiator (**right**). (**d**) Simple self-assembly schematic illustration of DNA nanoladders. One motif M1 and one motif M2 are highlighted.

An HCR (Figure 2a) was used to realize the response trigger of the structure, and T-junction cohesion (Figure 2b) was used to realize the connection of the structure together with an HCR. Based on this, our structure consists of three parts: two basic motifs and an initiator (Figure 2c). Each motif (M1 or M2) contains three single strands, forming a “Z”-shaped tile. Additionally, each motif contains three domains: two long arms and a central double helix domain. This central double helical domain (21 bp in length) serves as a rung and links the two arms, which is designed to consist of two shorter parts that hybridize complementarily with a longer part. One of these two long arms, the upper part of “Z” in Figure 2c, is called T-arm1 in M1 and T-arm2 in M2. The other long arm, the lower part of the “Z” in Figure 2c, is called H-arm1 in M1 and H-arm2 in M2. T-arm1 and T-arm2 are used for T-junction cohesion, and they contain a 26 bp DNA duplex and a 5 nt single-stranded overhang or bulge. The overhang and bulge of this arm have complementary sequences, so the “Z”-shaped upper halves of the two motifs are physically connected. H-arm1 and H-arm2 are used for an HCR, and each arm has a hairpin design with a 17 bp duplex as a stem, a 9 nt toehold, and a 9 nt loop. Each arm can be opened by a 9 nt long toehold-mediated strand displacement reaction, which changes the conformation of the motif. Therefore, the two long arms can hybridize with the corresponding domains of the adjacent motifs through complementary fragments, and they form periodic patterns. The initiator is complementary to the partial sequence of the hairpin arm (H-arm1) in motif M1 (a fragment composed of the toehold and stem), so that the toehold can open the hairpin arm of M1, thereby initiating the assembly.

The assembly process is carried out under isothermal conditions (Figure 2d). Triggered by the initiator, the conformation of M1 changes and the hairpin opens. At this time, a complex composed of the initiator and M1 (i.e., initiator·M1) is produced, which carries a reactive terminus, that is, the opened hairpin arm of M1. Then, the reactive end of this complex opens the hairpin arm of M2, and the opened hairpin arm continues to open the hairpin arm of M1. This process enables the realization of an HCR. An HCR makes the two hairpins hybridize into a double helix, which sterically promotes the stabilization of the 5 nt long T-junction cohesion. In this way, a pseudo-continuing DNA duplex structure is formed on both sides of the central helical domain, and motifs M1 and M2 alternately appear in a periodic pattern (in the nanoladder in Figure 2, M1 and M2 are represented in blue and red, respectively). Thus, a ladder-like DNA nanostructure is assembled. In the absence of the initiator, the hairpin arms of motifs M1 and M2 have a topological constraint, which prevents an HCR from occurring between the hairpin arms of M1 and M2. Without the assistance of the hairpin arm, the 5 nt long T-junction cohesion cannot exist stably in the solution, so M1 and M2 cannot further assemble into nanoladders. Therefore, only when the initiator is added to the mixture solution will the hairpin arms of M1 and M2 connect to each other and will the T-junction cohesion become stabilized, enabling the assembly to proceed.

### 2.2. Self-Assembly and Characterization of DNA Nanoladders

**Figure 3:**
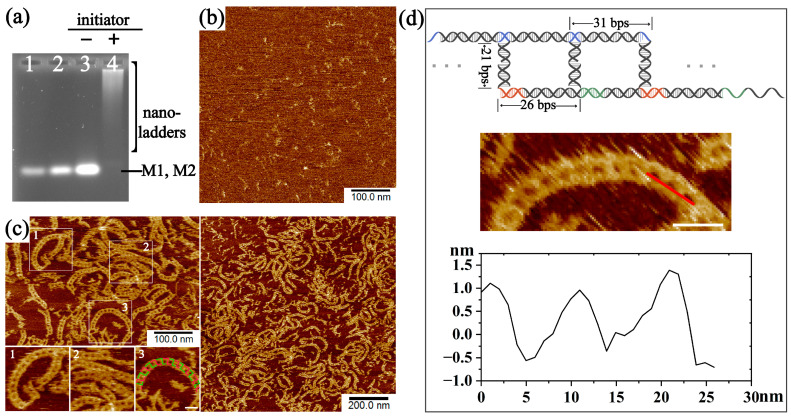
Result analysis of the self-assembly of DNA nanoladders. (**a**) Native agarose gel (1.5%) electrophoresis. The composition of each band is marked on the side of the gel. Lane 1 and lane 2 correspond to M1 and M2, respectively. Lane 3 represents the situation where the mixture solution of M1 and M2 reacts for a period of time without the initiator. Lane 4 represents the situation where the mixture solution of M1 and M2 react for a period of time with the initiator. With or without the initiator, the mixture solution reacts at room temperature (25 °C) for 16 h. The initiator and motif react at a molar ratio of 1:10. [M1] = [M2] = 250 nM, [initiator] = 25 nM. (**b**) AFM image of DNA nanoladders in the absence of initiator. [M1] = [M2] = 500 nM. (**c**) AFM images of DNA nanoladders in the presence of initiator. Three close-up views of the AFM image are shown. Scale bar: 20 nm. Initiator and motif react at a molar ratio of 1:10. [M1] = [M2] = 500 nM, [initiator] = 50 nM. (**d**) Schematic illustration and enlarged view of Figure 3c1. The inset represents the cross-sectional height of the red solid line in the AFM image. More cross-sections can be found in Supplementary Materials Section S5. All the scanned samples were diluted to 200 nM. Scale bar: 25 nm.

Motifs M1 and M2 were first pre-assembled by annealing with equal amounts of three single strands. Agarose gel electrophoresis analysis showed a high rate of a basic motif formation (clear single bands in lane 1 and lane 2 can be observed in Figure 3a). When the initiator was not added to the mixture solution of M1 and M2, M1 and M2 were not consumed after the reaction at room temperature, and no macromolecular structures were observed in the gel (lane 3 in Figure 3a). When the initiator was added to the mixture solution, after it reacted with M1 and M2 at a molar ratio of 1:10:10 at room temperature (25 °C) for 16 h, agarose gel electrophoresis showed that M1 and M2 were almost completely consumed, and a series of macromolecule structures were formed, which appeared as smears in the gel (lane 4 in Figure 3a). This result was consistent with our expected design results. When scanned with atomic force microscopy (AFM), in the absence of the initiator, no assembled DNA nanoladders were observed (Figure 3b). In the presence of the initiator, DNA nanoladders of different lengths randomly distributed on the mica surface were observed in the AFM image, and the pore morphology of the ladders could be clearly observed in the high-resolution image (Figure 3c and Figure S2). The length of the nanoladder measured in the gray background in Figure 3c was 154.417 nm (the measured nanoladder contained 16 repeats of the distance between the adjacent rungs, see Figure S3 for details). In Figure 3d, the width of the nanoladder was measured to be 9.874 nm (the rung was 21 bps, corresponding to 7.14 nm, and the DNA duplex was about 2 nm, see Figure S4 for details), and the distance between the two adjacent rungs was about 9.9835 nm (Figure 3d, inset. T-arm1 and T-arm2 were 31 bps, corresponding to 10.54 nm, and H-arm1 and H-arm2 were 26 bps, corresponding to 8.84 nm; see Figure S4 for details).

### 2.3. The Effect of Incubation Time, Incubation Temperature, and Ratio on Assembly

**Figure 4:**
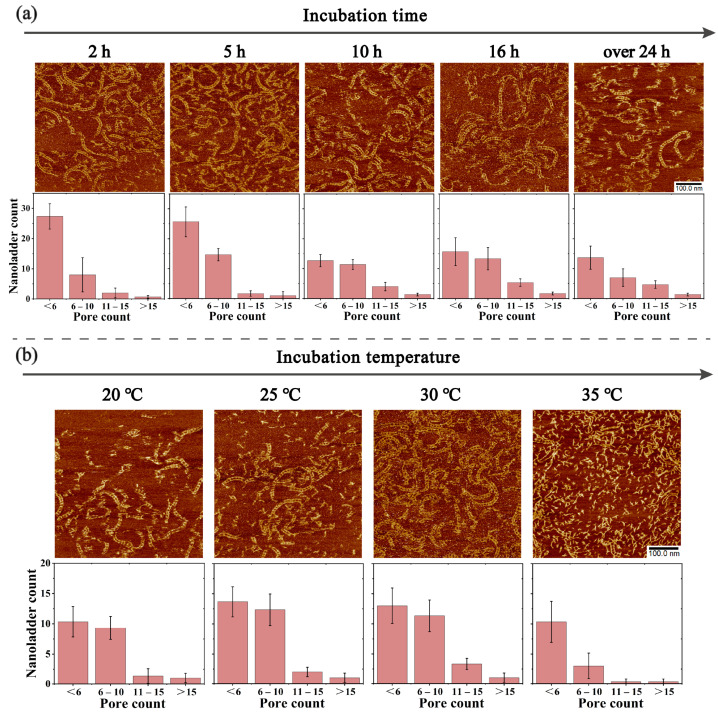
(**a**) AFM images and length statistics of DNA nanoladders at different incubation times. The lengths of nanoladders are divided into four groups (less than 6, 6 to 10, 11 to 15, and more than 15) according to the number of pores contained in the assembled structure. The x-axis of the statistical graph represents the groups, and the y-axis represents the number of nanoladders. The number of nanoladders in each group is marked above the bars. The initiator and motif are reacted at a molar ratio of 1:10. All experiments are performed at room temperature (25 °C) for different incubation times. (**b**) AFM images and length statistics of DNA nanoladders at different incubation temperatures. The initiator and motif are reacted at a molar ratio of 1:10. All experiments are performed for 16 h for different incubation temperatures. All the scanned samples were diluted to 200 nM. All the data in the bar chart are repeated three times. [M1] = [M2] = 500 nM, [initiator] = 50 nM.

After the characterization of the assembly nanoladders, we further investigated the formation of nanoladders under different self-assembly conditions. First, we explored the effect of different incubation times on the assembly. After the initiator and motif were mixed uniformly according to a molar ratio of 1:10, the mixture solution reacted for 2 h, 5 h, 10 h, 16 h, and over 24 h, and the products were characterized by AFM (Figure 4a and Figure S5). In Figure 4a, the incubation time increases from left to right, and the ladder-like structures observed in the scanning field at these five time points are pictured. The lower side in Figure 4a corresponds to the statistics of the length of nanoladders formed under different incubation times. The lengths of the nanoladders are represented and compared by the number of pores included (see Figure S6 for details), which are divided into four groups (first group: less than 6, second group: 6 to 10, third group: 11 to 15, and fourth group: more than 15). The statistics of the average length of the nanoladders at these five time points showed that with the increase in the incubation time, the average length of the nanoladders increased from 72.882 nm to 89.455 nm, which indicated that the average length of the nanoladders was positively correlated with the incubation time within a certain range. Next, we further analyzed the lengths of the nanoladders at different times according to the four groups. It can be seen from the statistical graph that after 2 h of incubation, the length of most of the nanoladders was in the first group, that is, the number of pores contained in the nanoladders was less than 6, indicating that the length of the nanoladders formed by incubation for 2 h is generally short. Starting from 5 h, the numbers of nanoladders in the first group and the second group were almost the same, and their sum total accounted for more than half of the total number of nanoladders in all groups, whereas the number of nanoladders in the fourth group accounted for about one tenth of the total number. The average length of the nanoladders in the fourth group exceeded 180 nm. After 16 h, the average length exceeded 190 nm, about 19 pores in length, which conformed to a reaction ratio of the initiator to the motif of 1:10. Interestingly, in the fourth group at 2 h, a small amount of nanoladders were generated with an average length of 181.703 nm, about 18 pores in length, which indicated that longer nanoladders can partially form and reach the length limit in a relatively short time, and the number of assembled structures increased with the extension of the incubation time.

In addition to the incubation time, the incubation temperature also has an effect on the assembly of the nanoladders. In the room temperature range, nanoladders can be assembled and remain stable; as the temperature increases, the nanoladders assembly gradually decreases to none. In previous experiments, the incubation of the nanoladders was performed at room temperature (25 °C). Here, we chose to test incubation temperatures above 5 °C and below room temperature, as well as 35 °C, 40 °C, and 45 °C. After the initiator and motif were uniformly mixed according to a molar ratio of 1:10, the mixture solution was incubated at different temperatures for 16 h and then analyzed by AFM (Figure 4b and Figure S7). In Figure 4b, the incubation temperature gradually increases from left to right. The AFM images show that the nanoladders formed well at 20 °C, 25 °C, and 30 °C without significant differences between them. The statistics showed that the number of nanoladders in the first group was almost the same as the number of nanoladders in the second group. The number of nanoladders in the fourth group accounted for about one tenth of the total, and their average length exceeded 194 nm. The length statistics at these three temperatures were similar, which was consistent with the corresponding AFM comparisons. When the incubation temperature increased to 35 °C, only a few short ladder-like structures could be seen in the AFM visual field, and the distribution in the visual field was very scattered. The statistical analysis showed that most of the nanoladders formed were short in length, and almost no longer structures were formed. However, when the temperature reached 35 °C, the average length of each group was almost the same as that of the same groups at other temperatures. Considering the proportion of each group, it can still be concluded that the assembly effect was greatly reduced at 35 °C. At 40 °C and 45 °C, no nanoladders were observed (not listed, see Figure S8 for details). There was no difference in the assembly effect of DNA nanoladders in the range of 20 °C to 30 °C. In this range, longer structures and clear pores were observed, indicating that the nanoladders remain stable at room temperature.

**Figure 5:**
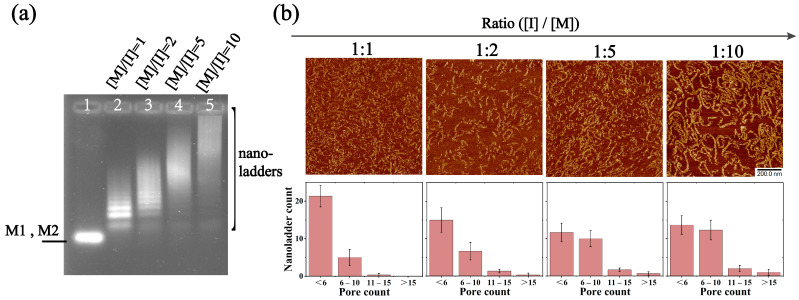
Analysis of different molar ratios between the initiator and assembly motifs M1 and M2. (**a**) Native agarose gel (1.5%) electrophoresis of different molar ratios between initiator and assembly motifs. The composition of the lanes is marked on the sides of the gel, and the molar ratio of the motif to the initiator corresponding to each lane is marked at the top of each lane. Lane 1 indicates that in the absence of the initiator, M1 and M2 are mixed in equal proportions and then spotting is performed immediately. Lane 2 to lane 5 represent the assembly of initiator and motif at different molar ratios. The concentrations of M1 and M2 are both 250 nM, and varying concentrations of initiator are added according to the indicated molar ratio. (**b**) AFM images and length statistics of DNA nanoladders. The proportion of the initiator gradually decreases from left to right. The concentrations of M1 and M2 are both 500 nM, and varying concentrations of the initiator are added according to the indicated molar ratio. All the scanned samples were diluted to 200 nM. All the data in the bar chart are repeated three times. For gel electrophoresis and AFM, all ratios of the reaction solutions are reacted at room temperature (25 °C) for 16 h.

When the DNA nanostructures are used as drug carriers, they need to be decorated with functionalities (e.g., targeting moieties and drugs) and used in different applications. For example, aptamer-tethered DNA nanoassemblies within 100 nanometers are highly desirable for biomedical applications [21]. Therefore, the size of the DNA nanostructure should be considered in practical applications and meet different needs. In this research, the size of the nanoladder can be adjusted by changing the ratio of the initiator to the substrates, which can then be selected on demand. At the same time, if there are higher requirements for size, a further size control can also be carried out through the purification method. The length of the nanoladders is negatively correlated with the molar ratio of the initiator and motif, so we explored the assembly results with the initiator and motif at different molar ratios. As the proportion of the initiator gradually decreases, the length of the nanoladders gradually increases. To this end, we first conducted agarose gel electrophoresis experiments. In Figure 5a, the mixture solution of M1 and M2 was spotted immediately after equal proportion mixing and a single band was present in lane 1. Gel electrophoresis showed that when the proportion of the initiator gradually decreased (that is, the molar ratio of the motif to initiator [M]/[I] gradually increased), the band migration speed of the corresponding lane gradually slowed down, indicating that the molecular weight of the formed structure gradually increased. In lanes 2 through 5, neither M1 nor M2 remained. Next, we used AFM for a further exploration. The AFM images (Figure 5b and Figure S9) clearly contrasted the relationship. The proportion of the initiator gradually decreases from left to right in Figure 5b. When the molar ratio of the initiator to the motif changed from 1:1 to 1:2, the number of nanoladders in the first group decreased, the number in the second group increased, and there were almost no nanoladders in the third or fourth groups. At these two ratios, the average length of the nanoladders changed from 44.929 nm to 53.016 nm. When the ratio changed to 1:5, the number of nanoladders in the first group further decreased, and the numbers in the third and fourth groups increased, whose sum total accounted for a quarter of the total statistical number. At this time, the average length was 78.673 nm. When the ratio changed from 1:5 to 1:10, the number of nanoladders in the first group decreased slightly and it was almost the same as the number in the second group, whereas the numbers of nanoladders in the third and fourth groups did not significantly change, which indicated that the self-assembly length of the nanoladders had a certain limit. When the molar ratio of the initiator and the motif was 1:10, the average length of the assembled nanoladders was 84.663 nm, which was quite different from the expected length of the designed self-assembly, indicating that only some of the nanoladders were assembled according to the designed scale. The above results may be related to the tile structure we designed. Additionally, the assembled nanoladder has a certain degree of curvature, so when the cumulative curvature of the nanoladder reaches a certain value, the T-junction cohesion may become unstable, thereby hindering the further growth of nanoladders or causing longer nanoladders to break.

### 2.4. DNA Nanoladders Serve as Structural Scaffolds with Biomolecules

**Figure 6:**
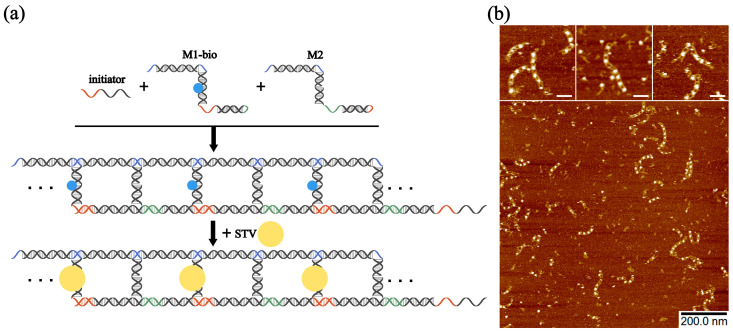
(**a**) Simple self-assembly schematic illustrations of motif M1-bio, M2, and initiator. Motif M1-bio is modified with biotin first (blue dots represent biotin) and then labeled with streptavidin (STV) (yellow spheres represent STV) after assembly. (**b**) AFM images of the STV-decorated nanoladders. Three close-up views of the AFM images are shown. Scale bar: 20 nm. [M1-bio] = [M2] = 500 nM, [initiator] = 50 nM. The final concentration of the assembled nanoladders is 500 nM, and the concentration of STV is 5 µM.

Recently, it has been proven that assembled DNA nanostructures can be used as versatile structural scaffolds [43,45], providing new methods for drug delivery, molecular machines, and other applications. Biotin molecules can be used to modify DNA nanostructures. Because of the specific binding capacity of biotin to streptavidin (STV), STV can be precisely bound to biotin modification sites, which enables the precise localization of the biomolecules. As shown in Figure 6a, the 5′end of the rung of the motif M1-bio (whose DNA sequence was identical to motif M1) was modified with biotin. After being triggered by the initiator, motifs M1-bio and M2 assembled into DNA nanoladders modified with biotin. Due to the alternate assembly of M1-bio and M2, the biotin molecules were spaced along the nanoladders. Thereafter, a solution of biotin-modified nanoladders was supplemented with a ten-fold aqueous STV solution, and the reaction continued for ten minutes at room temperature. The STV molecules bound to the modification site of biotin, located in the horizontal intermediate position of the nanoladder, and STV were also distributed across the nanoladders at intervals. The AFM images showed white bright spots which were spaced in ladder-like structures (Figure 6b), and this result was consistent with the design. Further studies can use motif M2 for specific binding or to try different modified objects such as nanoparticles and colloidal particles, to expand the role of the functional scaffolds of nanoladders.

## 3. Materials and Methods

### 3.1. Materials

The designed DNA sequences were synthesized and purified by GenScript Biotech Co., Ltd. (Nanjing, China). DNA strands modified with biotin were purified by High Performance Liquid Chromatography (HPLC), and other strands were purified by economic PAGE (ePAGE) or PAGE. The DNA strands are shown in Figure S1. All sequences are shown in Table S1. The concentration of each strand was estimated by measuring the UV absorbance at the wavelength of 260 nm using a NanoDrop 2000 spectrophotometer (Thermo Fisher Scientific Inc., Waltham, MA, USA). STV (used without further purification), agarose, and GelRed were purchased from Sangon Bio-tech Co., Ltd. (Shanghai, China). NiCl_2_ was purchased from Sinopharm Chemical Reagent Co., Ltd., Shanghai, China. All aqueous solutions were prepared with ultrapure water.

### 3.2. Preparation of Basic Motifs

The buffer we used contained 40 mM of Tris (pH 8.0), 20 mM of acetic acid, 2 mM of EDTA, and 12.5 mM of magnesium acetate. DNA single strands were dissolved in 1 × TAE-Mg^2+^ buffer. To prepare motif M1 and M2, all strands were equivalently mixed at the concentration of 4 µM. Then, the mixture solution was annealed to assemble the motifs, heated at 95 °C for 5 min, and held at 65 °C for 30 min, 50 °C for 30 min, 37 °C for 30 min, and 25 °C for 30 min.

### 3.3. Self-Assembly of DNA Nanoladders

The assembled M1 and M2 were equivalently mixed in 1 × TAE-Mg^2+^ buffer. Then, 0.5 µL of initiator solution was added to 40 µL of the mixture solution (final molar ratio: [M]/[I] = 10). The mixture was incubated at room temperature (25 °C) for 16 h.

### 3.4. Agarose Gel Electrophoresis

The samples were run on 1.5% native agarose gel at a constant voltage of 65 V for 90 min at 4 °C. The running buffer was 1 × TAE-Mg^2+^ buffer. The electrophoresis equipment was a mini-sub cell GT electrophoresis unit (Bio-Rad Laboratories, Inc., Hercules, CA, USA). After electrophoresis, the gel was stained with GelRed nucleic acid dye and scanned with a Molecular Imager Gel Doc XR+ imaging system (Bio-Rad).

### 3.5. Biotin–STV Binding Nanoladders

After the biotin-labelled DNA nanoladders were assembled, STV in 1 × TAE/Mg^2+^ buffer was added, and the finial molar ratio of the STV to biotin-labelled DNA nanoladders was 10:1. The mixture was left at room temperature for 10 min and then characterized by AFM.

### 3.6. AFM Imaging

For AFM imaging, 20 µL of NiCl_2_ (1 mM) was deposited onto a freshly cleaved mica surface (Ted Pella, Inc., Redding, CA, USA) and left to adsorb on the surface for 5 min. Then, the surface was washed three times with ultrapure water. A drop of 2.5 µL of the DNA sample was deposited onto the mica surface and left to adsorb for another 5 min. Then, the surface was washed with 1 × TAE/Mg^2+^ buffer to remove the excess DNA. Next, 20 µL of 1 × TAE/Mg^2+^ buffer was added to the mica surface. Imaging was performed in a fluid cell in ScanAsyst in the fluid mode on a Multimode 8 AFM (Bruker Scientific LLC., Billerica, MA, USA) with ScanAsyst-fluid + probe (Bruker).

## 4. Conclusions

In summary, we assembled a ladder-like DNA nanostructure. The assembly process was initiated by trigger strands and then the basic motif assembled into periodic nanoladders. The rung of this nanoladder was flanked by a pseudo-continuous DNA double helix formed by T-junction cohesion and an HCR. After determining the best conditions, the nanoladders are imparted with the function of the drug carrier by adding drug loading sites. In addition to carrying drug loading sites (e.g., double-stranded 5′-GC-3′ or 5′-CG-3′ sequences for loading doxorubicin, DOX) to achieve a drug delivery in many ways (e.g., transfections and endocytosis), the DNA nanoladder we report can also be modified with fluorescent agents for bioimaging. In addition, due to the presence of an initiator, biosensing can also be carried out, which can not only recognize miRNAs but also recognize other molecules by conjugating aptamers to the initiator [21].

Including DNA nanoladders, DNA-based nanostructures are suitable for biomedical applications, such as in drug delivery, due to their low immunogenicity and low toxicity [46,47]. Moreover, in the serums, or even in the cellular milieu [48], they can maintain a certain structural integrity for a certain period of time so as to complete the task of drug delivery. This may be because the folding of DNA strands enables the structures to escape cellular DNase and possible physiological aggressors [47,49], achieving a greater stability than the unfolded ones. These characteristics have enabled DNA carriers to develop effectively in the biomedicine arena.

Undoubtedly, despite all the advantages and expectations, the DNA nanoladder we reported still has a lot of limitations. One limitation is the DNA nanoladder’s dependence on metal ions, which makes it sensitive to the depletion of metal ions, so it is still a long way to go before it has a practical use. Another limitation is the high cost of the manufacture of the nanoladder at the gram or kilogram scale, making it difficult for them to be used in the clinic compared to common chemical drugs. In addition, there is a technical limitation, that is, it is not possible to produce large quantities of nanoladders with an accurate length in a single step. Although the nanoladder can be adjusted by the ratio of the initiator to substrates, the precise control of the size is very difficult to be carried out on the nanoladder. To obtain the nanoladder with an accurate length, a further purification is needed, which potentially increases the production cost of the nanoladder. Overall, we believe that our self-assembly system will provide new tools for the research of molecular machines, drug delivery, and biosensing.

## Data Availability

The data presented in this study are available on request from the corresponding author.

## Supplementary Materials

The following supporting information can be downloaded at: https://www.mdpi.com/article/10.3390/molecules28020797/s1. Figure S1: Schematic illustrations of motifs; Figure S2: Enlarged view of Figure 2c in the main text and an additional AFM image of DNA nanoladders; Figure S3: Steps for measuring ladder length in FIJI; Figure S4: Dimensional measurement of pores; Figure S5: Additional AFM images of DNA nanoladders at different incubation times; Figure S6: Counting process of nanoladder pores; Figure S7: Additional AFM images of DNA nanoladders at different incubation temperatures; Figure S8: AFM images of nanostructures incubated at 40 °C and 45 °C; Figure S9: Additional AFM images of DNA nanoladders at different ratios; Table S1: The DNA sequences used to assemble DNA nanoladders.

## Sample Availability

Samples of the compounds are not available from the authors.
