# Supplementary material for: Active Self-Assembly of Ladder-Shaped DNA Carrier for Drug Delivery
Source: Molecules. 2023 Jan 13;28(2):797. doi: 10.3390/molecules28020797 (PMC9862081; doi:10.3390/molecules28020797)
Supplement: Supplementary file 1 [file molecules-28-00797-s001.zip › molecules-2127399-supplementary.pdf]

Active self-assembly of ladder-shaped DNA carrier for drug delivery

Supplementary Material

Yuan Liu [1], Jiaxin Wang [2], Lijun Sun [2], Bin Wang [2], Qiang Zhang [1,*], Xiaokang Zhang [1], and Ben Cao [1]

[1] School of Computer Science and Technology, Dalian University of Technology, Dalian 116024, China

[2] Key Laboratory of Advanced Design and Intelligent Computing, Dalian University, Ministry of Education, Dalian 116622, China

* Correspondence: zhangq@dlut.edu.cn

1 DNA sequences

DNA sequences are designed with NUPACK to reduce undesired hybridization between DNA strands. DNA sequences are shown in Table S1.

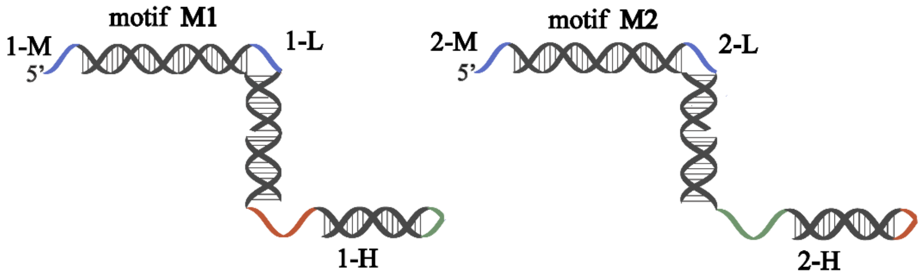

Figure S1. Schematic illustrations of motifs. The name of each strand is marked at the 5' end.

Table S1. The DNA sequences used to assemble DNA nanoladders

Name	DNA Sequence (5' to 3')
**1-M**	TGCCAGGCTTCGTGTTACATAGAGGGATAATGTGAACCTT
**1-H**	CCTCGTAGTCACGTTGAAGGTGGATGAGTGGAGTGAAGACCA TCCAACTTCACTCCACTCATCC

**1-L**	GTGACTGCGAGGAAGGTTCACTGGCAATTATCCCTCTATGTAACACGAAGCC
**2-M**	TGCCACCGTTCGTGTTACATAGAGGGATAATGTGAACCTTCCTCGTAGTCAC
**2-H**	CTTCACTCCACTCATCCACCTTCAACGGATGAGTGGAGTGAAGTTGGATGGTGTGACTGCGAGG
**2-L**	AAGGTTCACTGGCAATTATCCCTCTATGTAACACGAACGG
**initiator**	CTTCACTCCACTCATCCACCTTCAAC
**1-H-bio**	Biotin-CCTCGTAGTCACGTTGAAGGTGGATGAGTGGAGTGAAGACCATCCAACTTCACTCCACTCATCC

Complementary domains of T-junctions (single-stranded overhangs and bulges) are marked in blue.

The toehold of the hairpin arm of motif **M1** is marked in dark red, and the loop is marked in green.
The toehold of the hairpin arm of motif **M2** is marked in green, and the loop is marked in dark red.

2 Self-assembly and characterization of DNA nanoladders

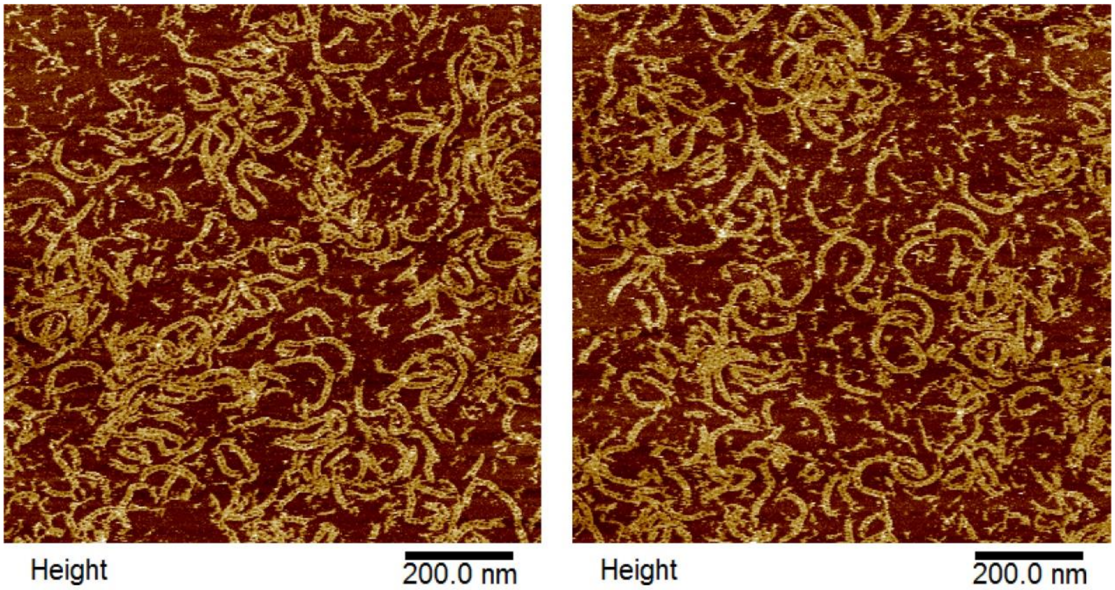

Figure S2. Enlarged view of Figure 2c in the main text and an additional AFM image of DNA nanoladders. Sample and reaction conditions are the same as in Figure 2c in main text. Initiator and motif react at a molar ratio of 1:10. All experiments are incubated at room temperature (25 °C) for 16 h. [M1] = [M2] = 500 nM, [initiator]=50 nM.

3 Measurement of nanoladder size

3.1 Measurement of nanoladder length

FIJI is easy to operate and can be used to measure the length of the curve. Here, we use this software to measure the length of the nanoladder.

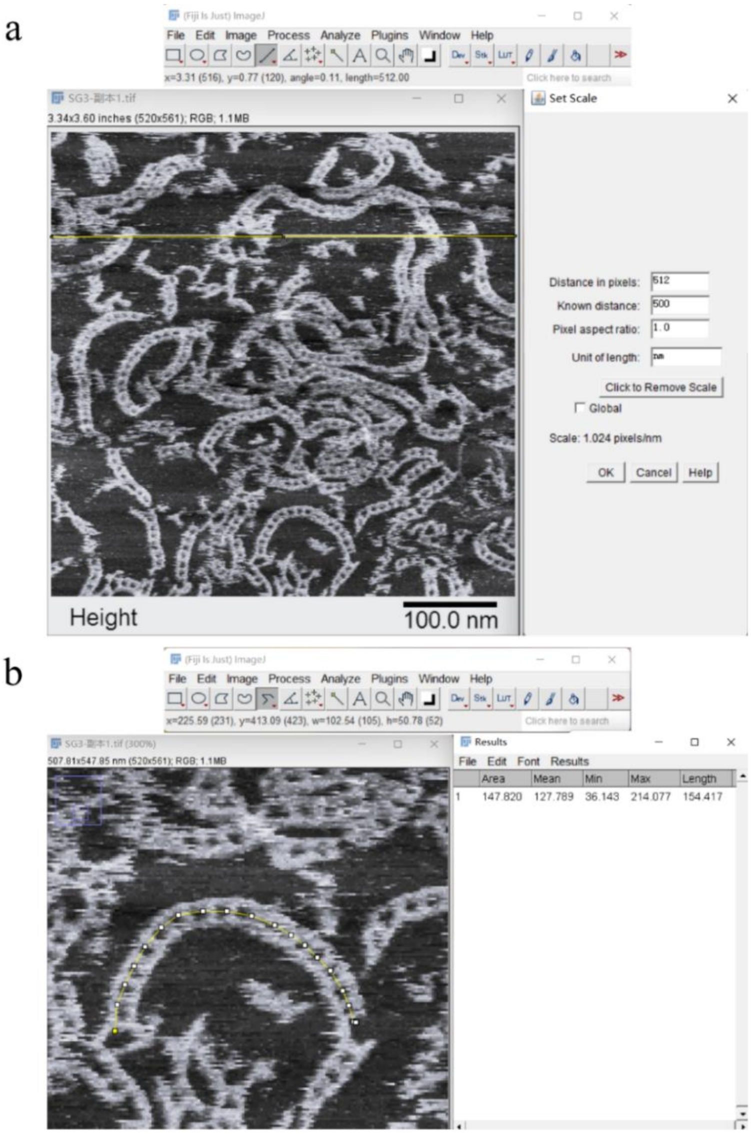

Figure S3. Steps for measuring ladder length in FIJI. (a) Set the length scale. Draw a “straight line” parallel to the horizontal edge of the image, then choose “Set Scale” from “Analyze” to set the length scale. (b) Measure the length of nanoladder. Draw a “Segmented line” along a DNA nanoladder, then choose “Measure” from “Analyze” to measure the length of the segmented line, which represents the length of the nanoladder.

The length of the nanoladder in **Figure S2b** was measured to be 154.417 nm. The measured nanoladder contains 16 repeats of the distance between the adjacent rungs, the distance is calculated to be 9.53 nm. The calculation process is as follows：(154.417 - 2）/ 16 = 9.53 nm.

3.2 Dimensional measurement of pores

We use NanoScope Analysis offline processing software to measure the width of the nanoladder and the distance between adjacent rungs.

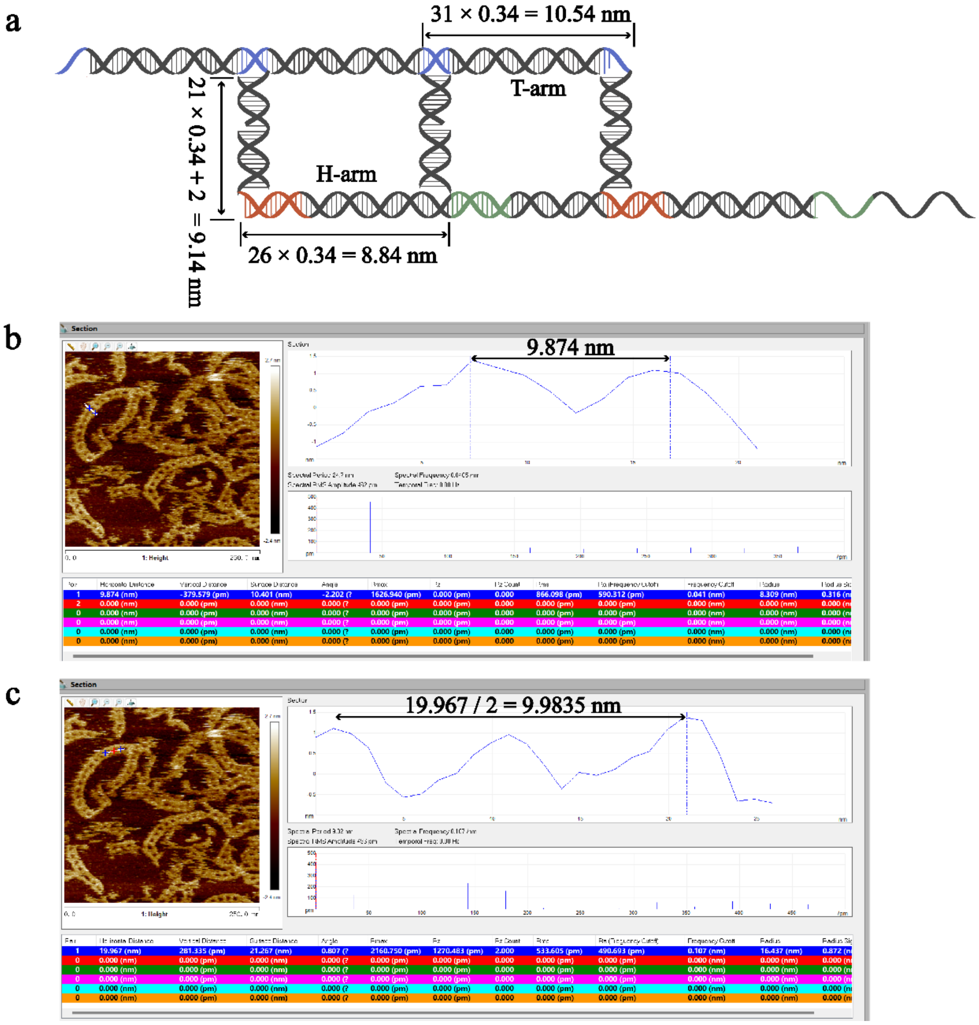

Figure S4. (a) Theoretical dimensions of DNA nanoladder. The theoretical lengths of T-arm and H-arm are 10.54 nm and 8.84 nm, respectively. The theoretical width of nanoladder is 9.14 nm. (b) The measured width of nanoladder is 9.874 nm. The measured value agrees with the theoretical value. (c) The measured distance between two adjacent rungs of the nanoladder is 9.9835 nm. The measured value agrees with the theoretical value.

The distance between adjacent rungs is measured to be 9.9835 nm, which is in agreement with the calculated value in **Figure S2**.

4 Additional AFM images of nanoladders under different self-assembly conditions

4.1 AFM images at different incubation times

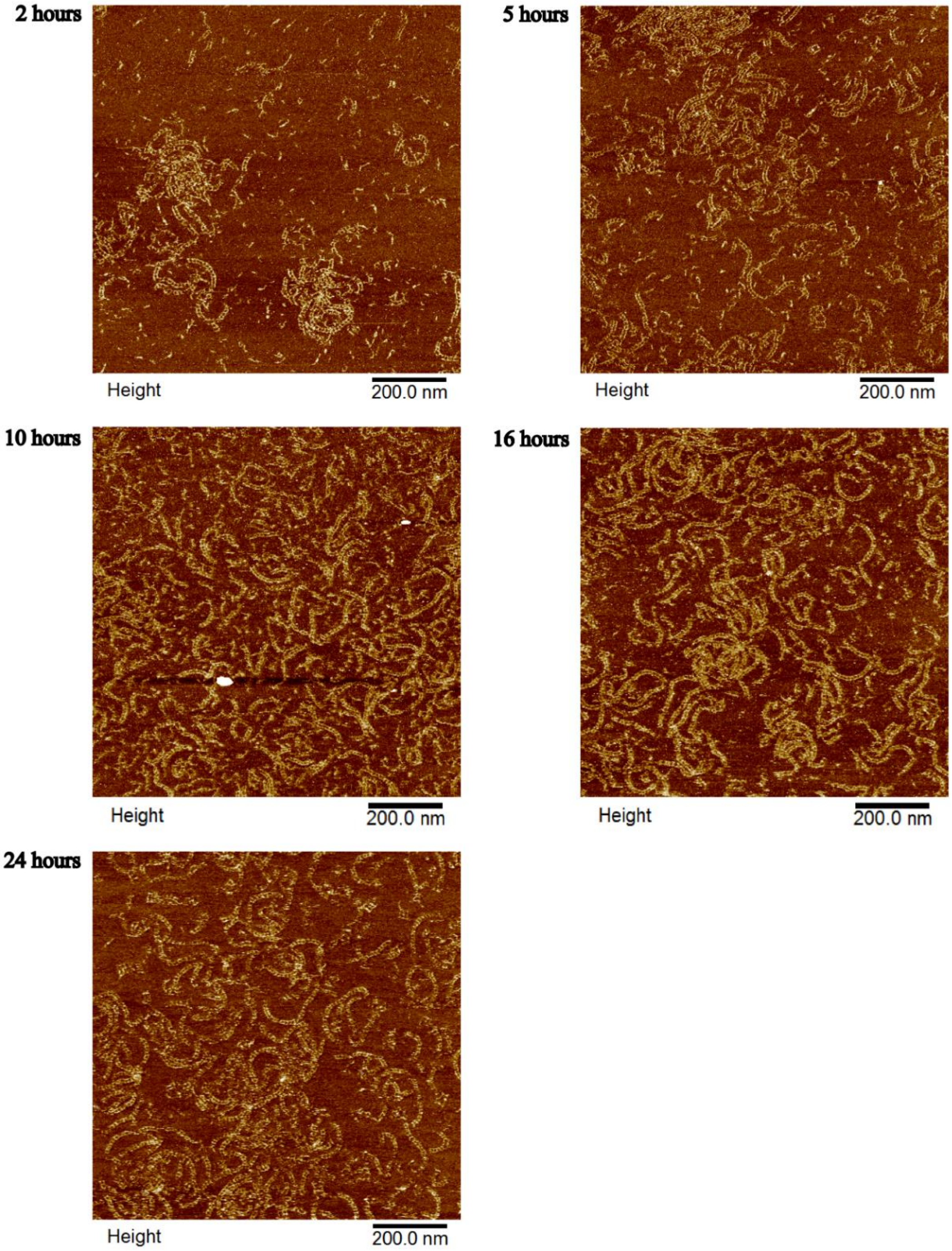

Figure S5. Additional AFM images of DNA nanoladders at different incubation times. Initiator and motif are reacted at a molar ratio of 1:10. All experiments are incubated at room temperature (25 °C) for different incubation times. [M1] = [M2] = 500 nM, [initiator] = 50 nM.

4.2 Indication and counting of pores

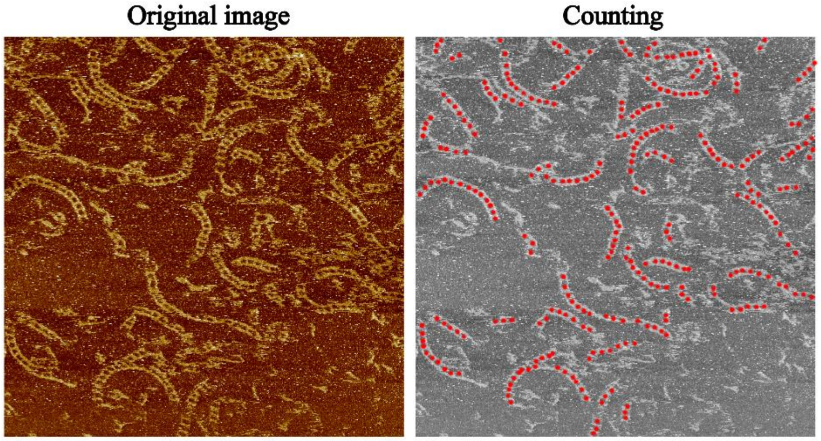

num	counted
<6	34（34%）
6~10	36（36%）
11~15	20（20%）
>15	10（10%）

Figure S6. Counting process of nanoladder pores. Take the AFM image on the left side of Figure 3a of the main text as an example. The red dots represent the pores contained in the nanoladders. All experiments are incubated at room temperature (25 °C) for 2 h. [M1] = [M2] = 500 nM, [initiator] = 50 nM. Image size: 500 × 500 nm.

4.3 AFM images at different incubation temperatures

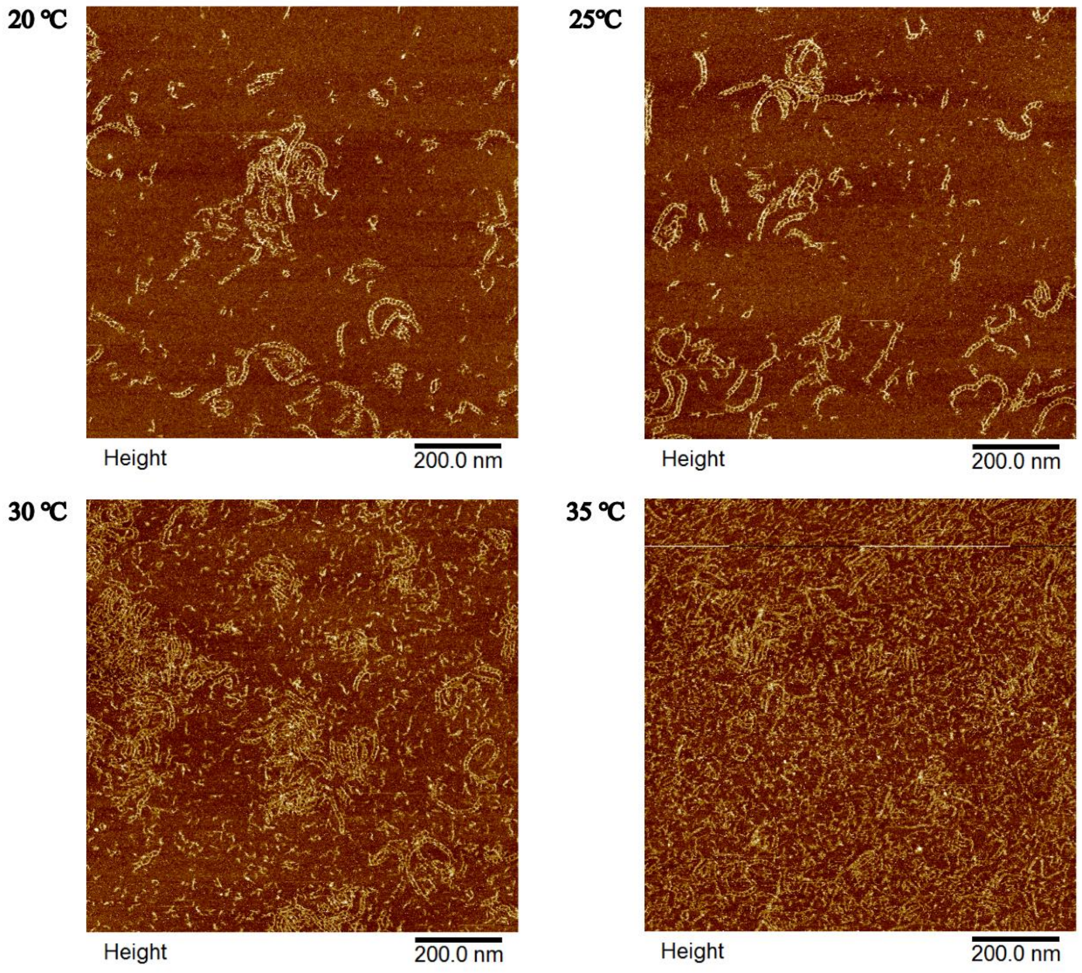

Figure S7. Additional AFM images of DNA nanoladders at different incubation temperatures. Initiator and motif are reacted at a molar ratio of 1:10. All experiments are incubated for 16 h at different incubation temperatures. [M1] = [M2] = 500 nM, [initiator] = 50 nM.

4.4 AFM images at other incubation temperatures

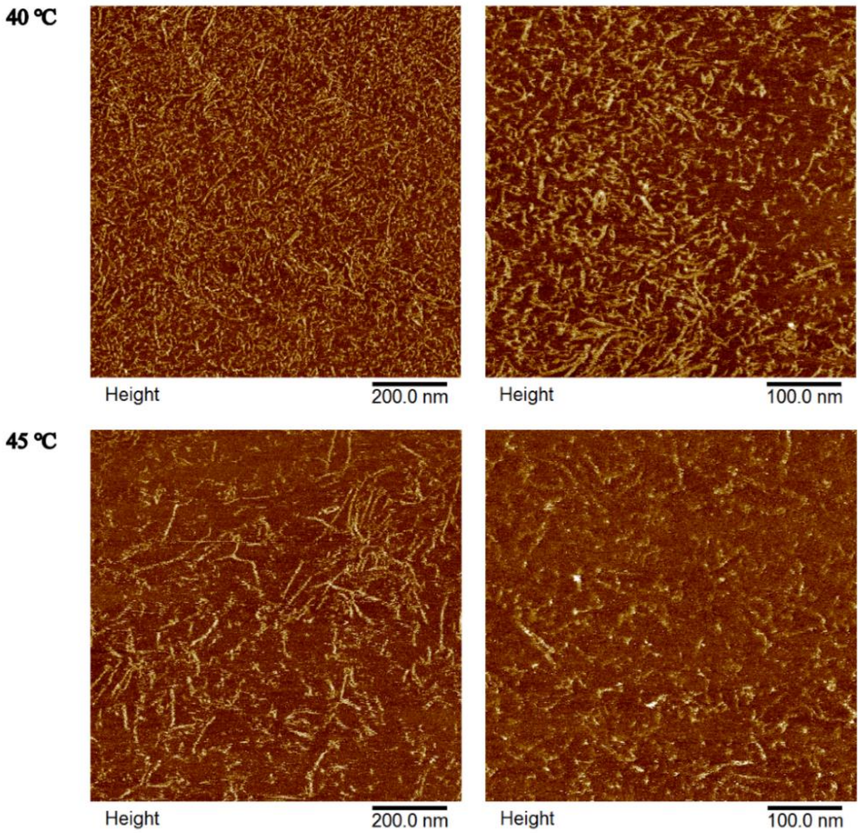

Figure S8. AFM images of nanostructures incubated at 40°C and 45°C. Initiator and motif are reacted at a molar ratio of 1:10. All experiments are incubated for 16 hours at different incubation temperatures. [M1] = [M2] = 500 nM, [initiator] = 50 nM.

At 40 °C and 45 °C, the pore morphology of nanoladders could not be observed in the scanning field, but some thin and long fibrous structures appeared.

4.5 AFM images at different ratios

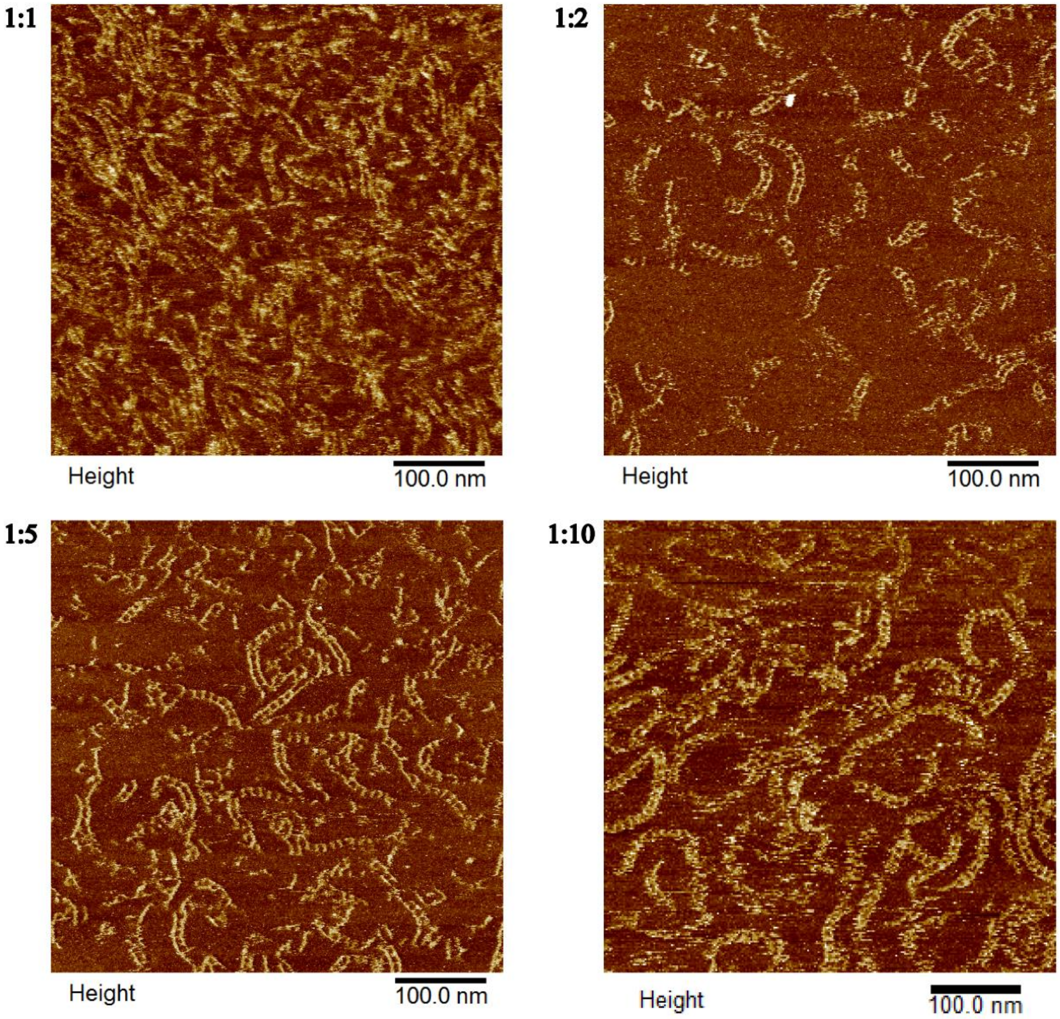

Figure S9. Additional AFM images of DNA nanoladders at different ratios. The concentrations of M1 and M2 are both 500 nM, and varying concentrations of initiator are added according to the indicated molar ratio. All ratios of reaction solutions are reacted at room temperature (25 °C) for 16 h.

5 Additional cross-sections

The curve chart represent the cross-sectional height of the green solid line in the AFM images.

1	2	3	4	5
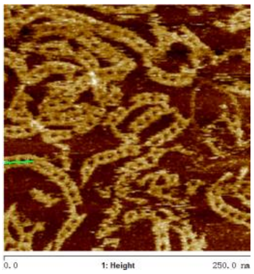	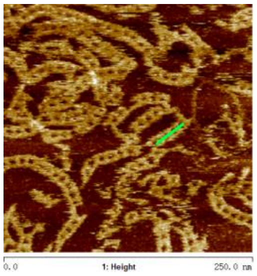	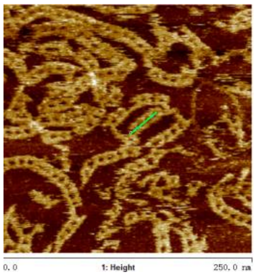	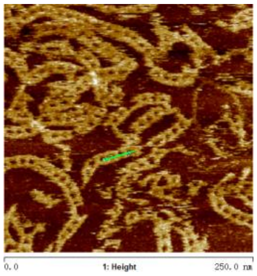	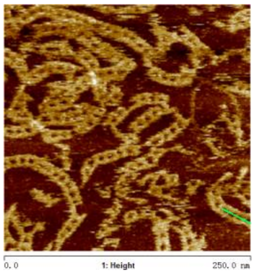
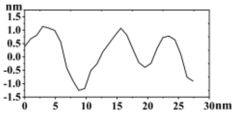	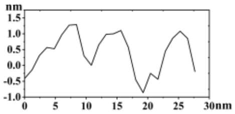	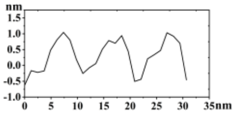	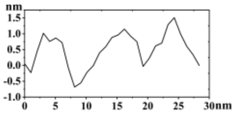	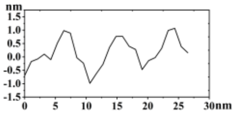
6	7	8	9	10
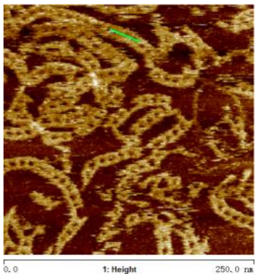	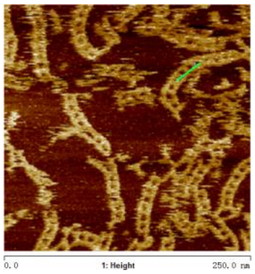	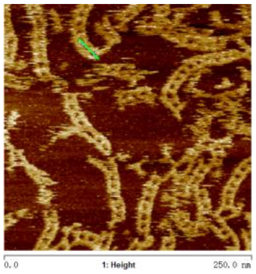	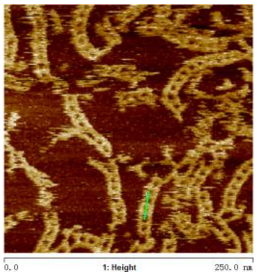	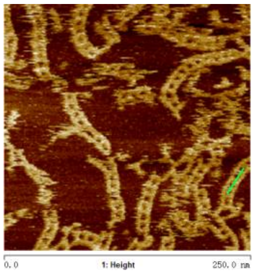
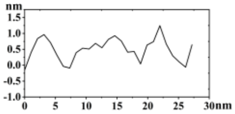	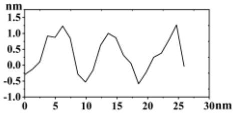	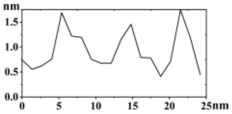	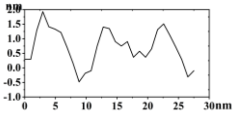	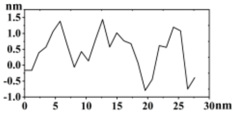
11	12	13	14	15
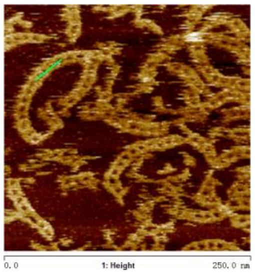	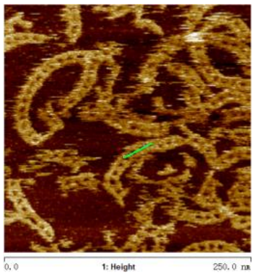	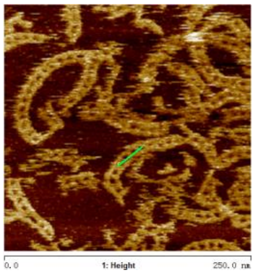	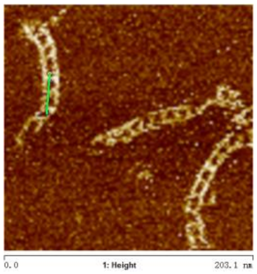	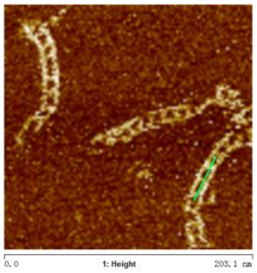
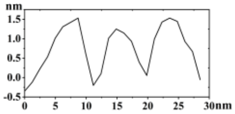	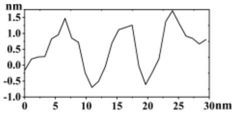	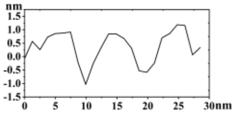	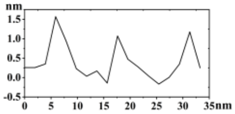	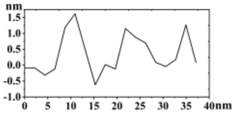

16	17	18	19	20
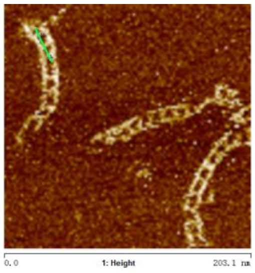	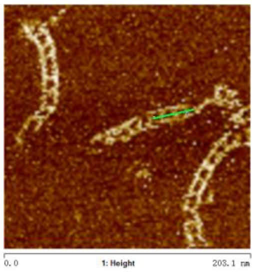	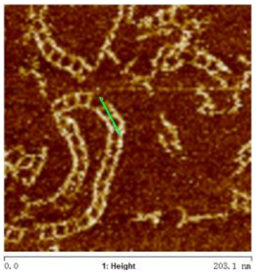	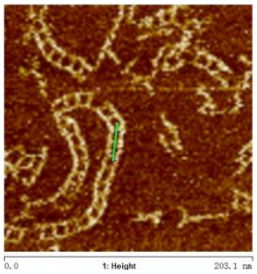	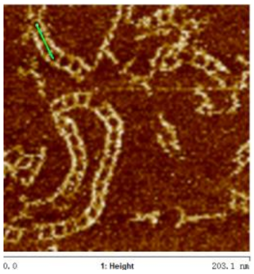
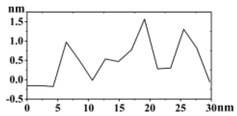	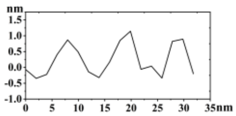	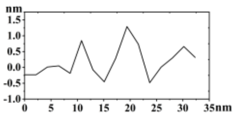	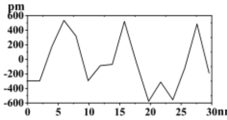	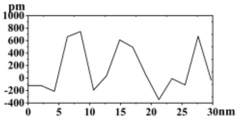
21	22	23	24	25
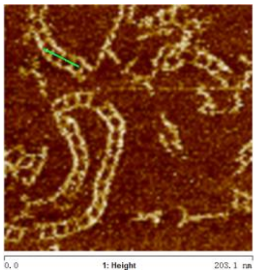	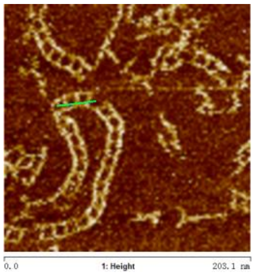	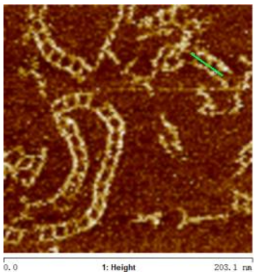	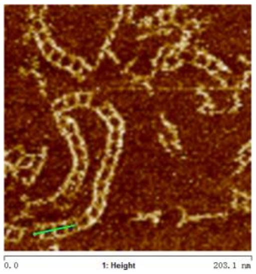	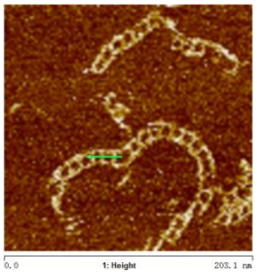
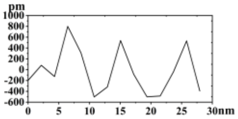	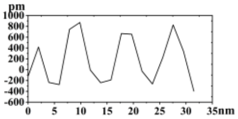	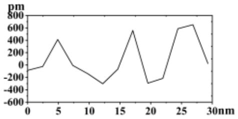	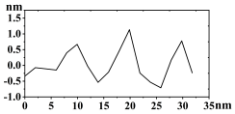	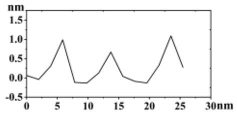
26	27	28	29	30
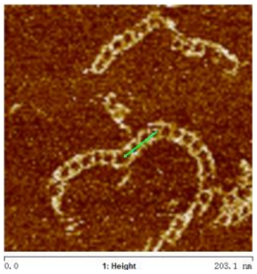	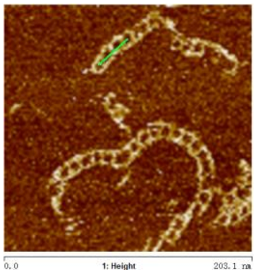	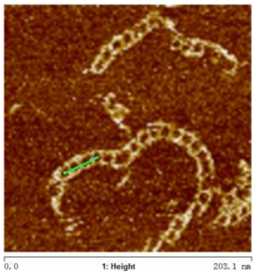	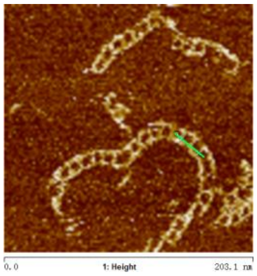	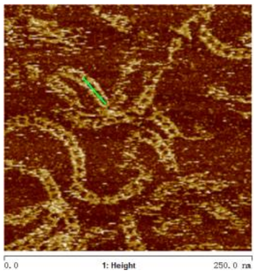
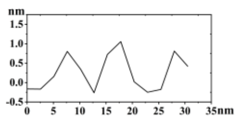	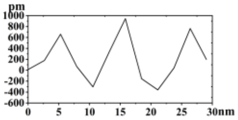	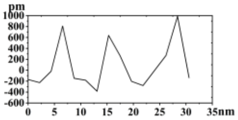	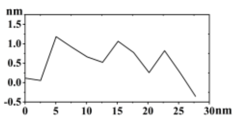	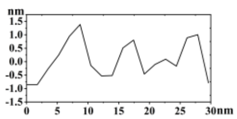

31	32	33	34	35
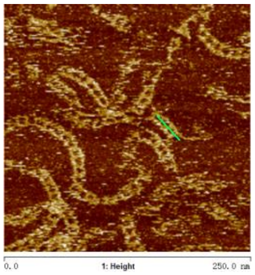	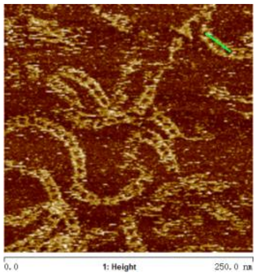	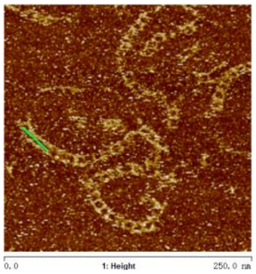	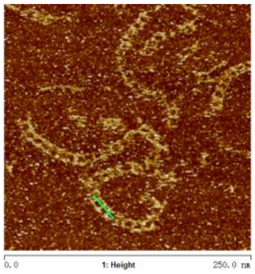	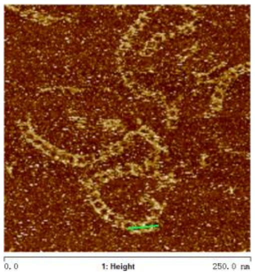
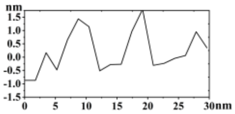	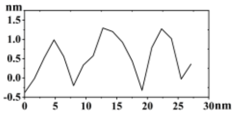	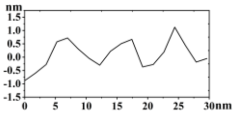	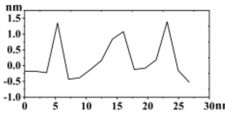	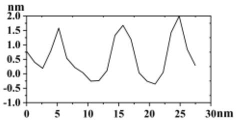
36	37	38	39	40
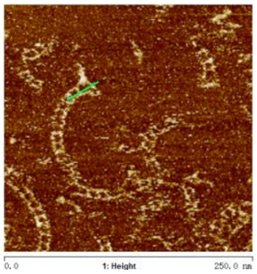	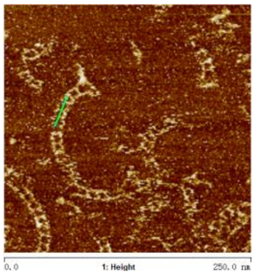	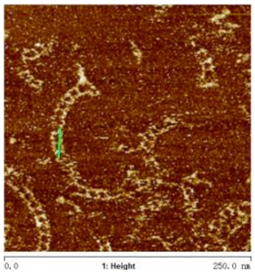	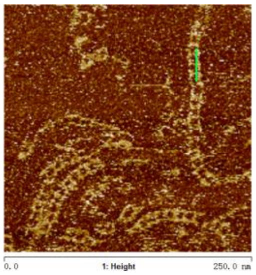	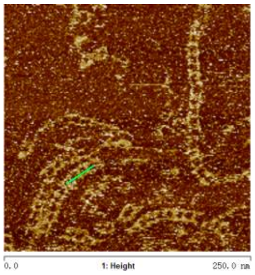
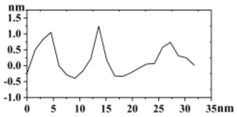	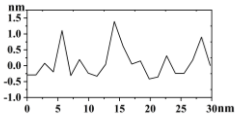	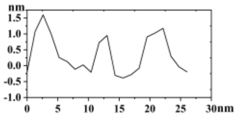	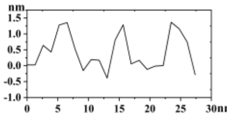	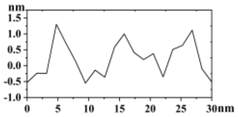
41	42	43	44	45
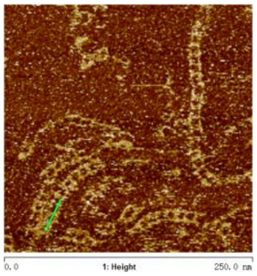	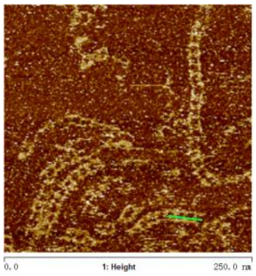	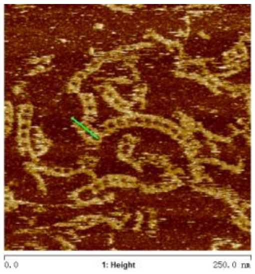	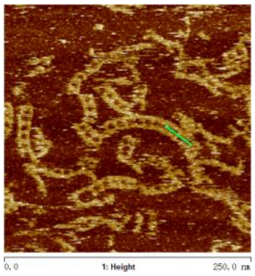	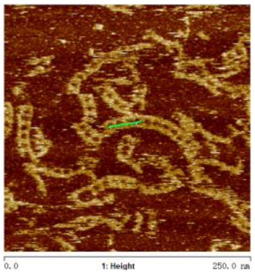
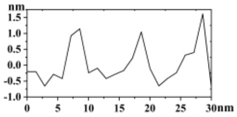	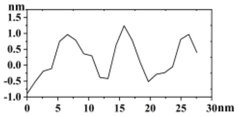	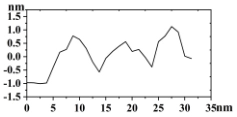	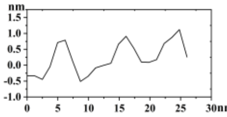	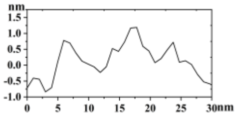

46	47	48	49	50
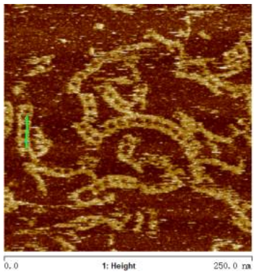	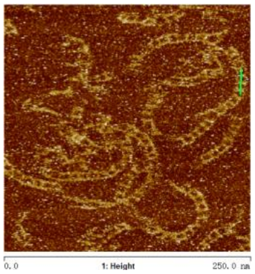	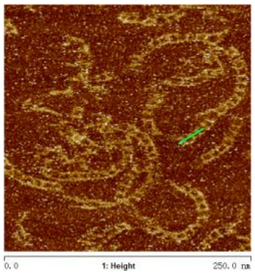	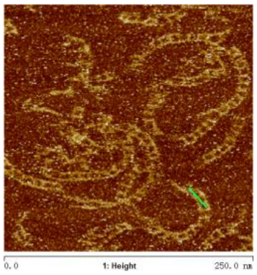	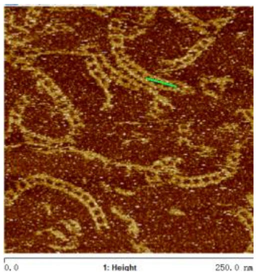
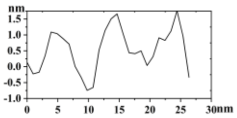	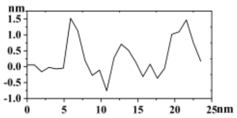	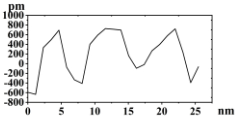	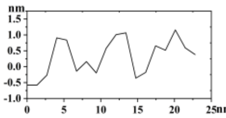	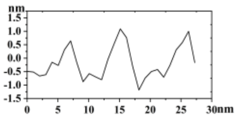
51	52	53	54	55
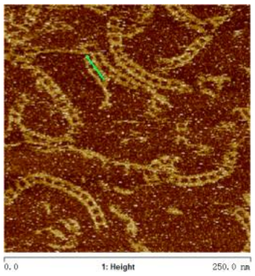	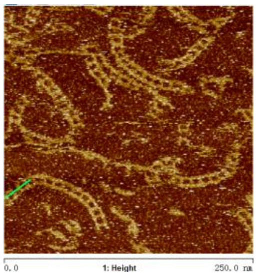	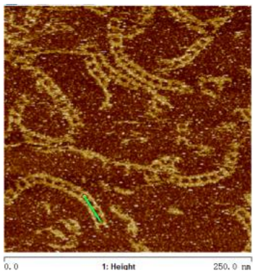	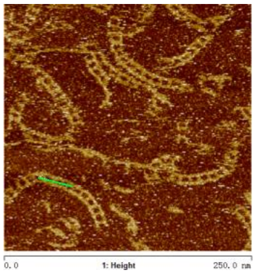	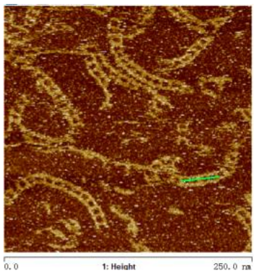
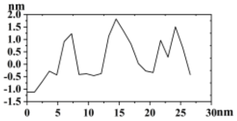	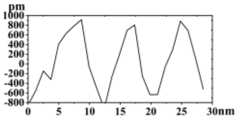	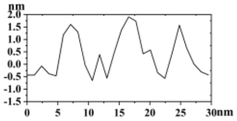	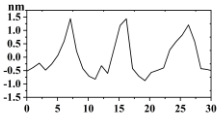	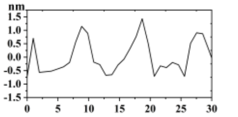
56	57	58	59	60
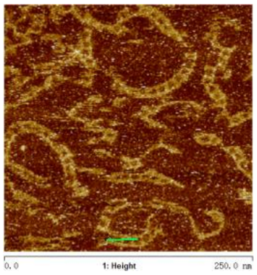	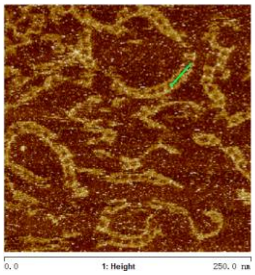	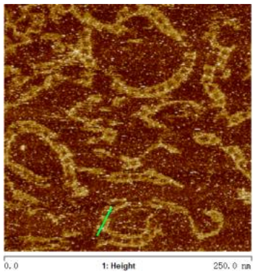	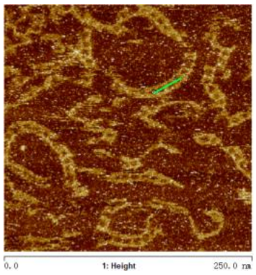	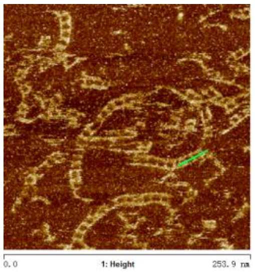
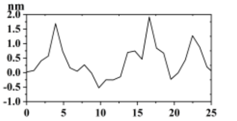	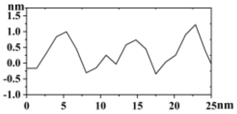	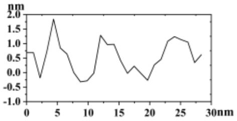	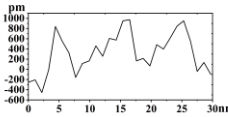	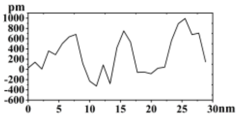

61

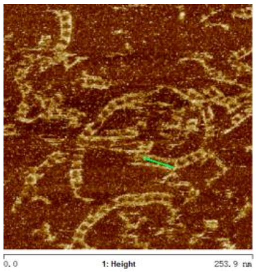

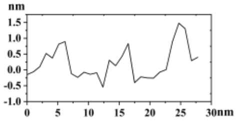

62

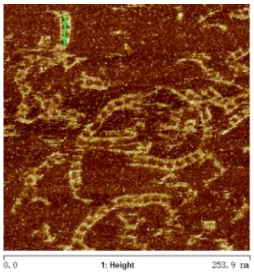

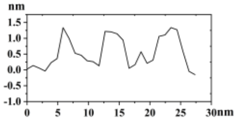

63

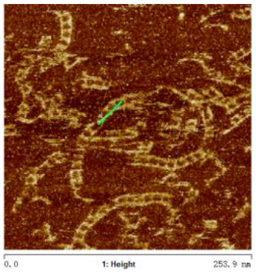

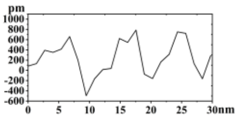

64

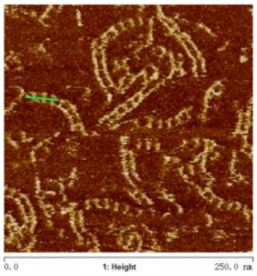

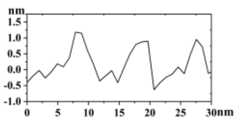

65

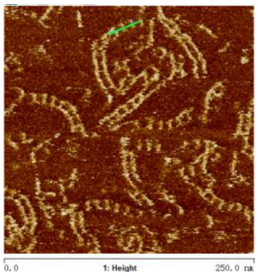

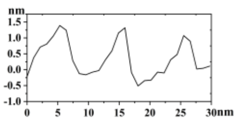

66

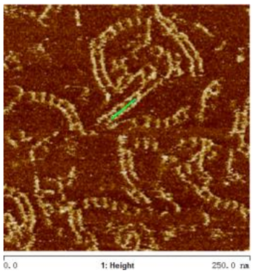

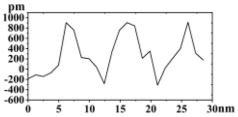

67

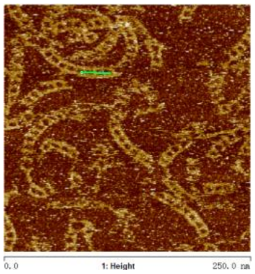

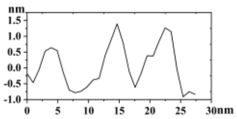

68

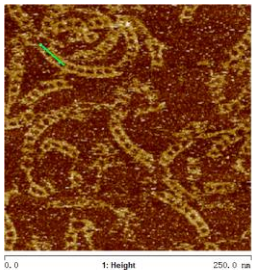

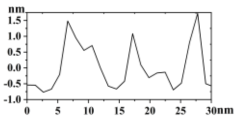

69

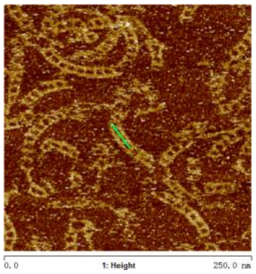

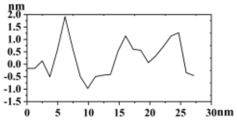

70

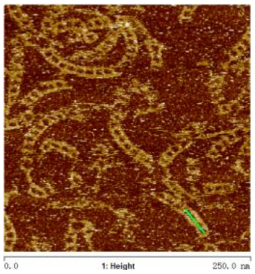

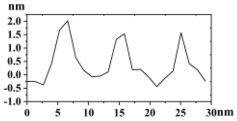

71

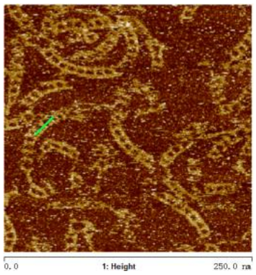

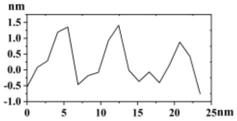

72

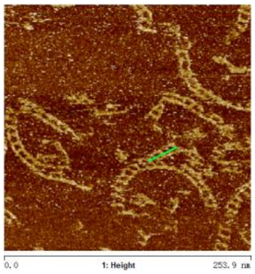

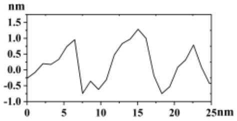

73

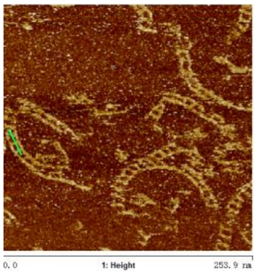

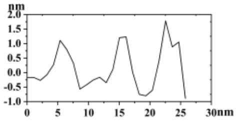

74

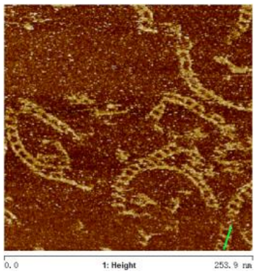

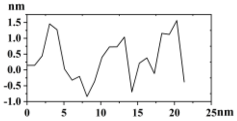

75

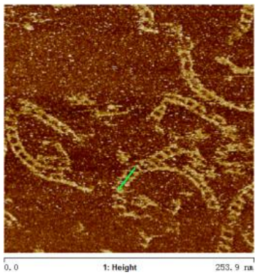

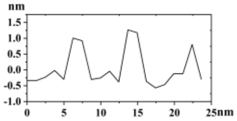

<table>
<tr><td>76</td><td>77</td><td>78</td><td>79</td><td>80</td></tr>
<tr>
<td>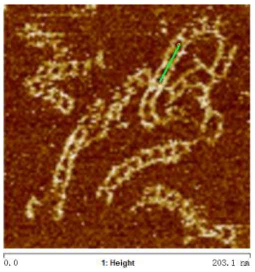</td>
<td>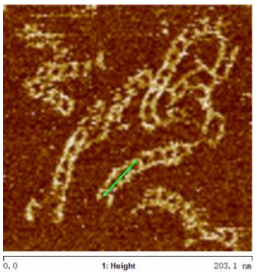</td>
<td>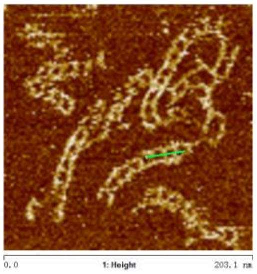</td>
<td>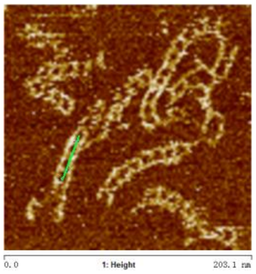</td>
<td>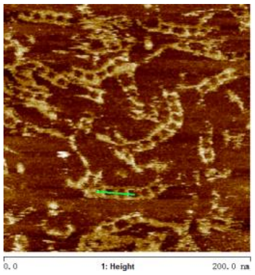</td>
</tr>
<tr>
<td>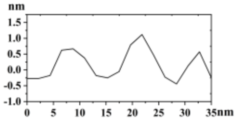</td>
<td>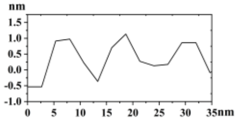</td>
<td>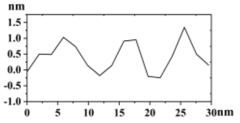</td>
<td>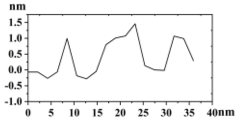</td>
<td>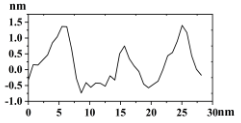</td>
</tr>
<tr><td>81</td><td>82</td><td>83</td><td>84</td><td>85</td></tr>
<tr>
<td>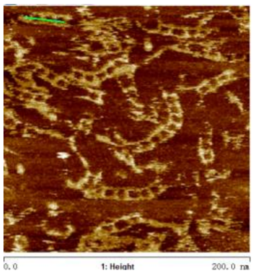</td>
<td>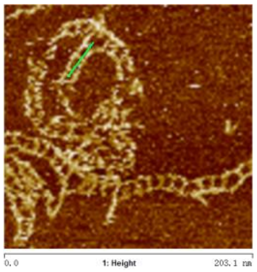</td>
<td>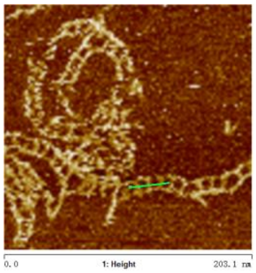</td>
<td>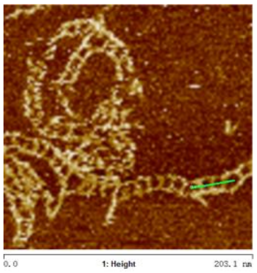</td>
<td>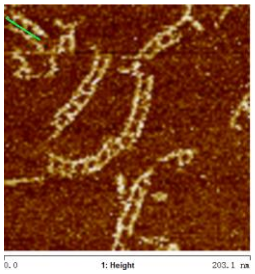</td>
</tr>
<tr>
<td>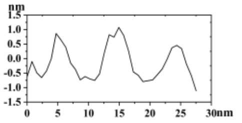</td>
<td>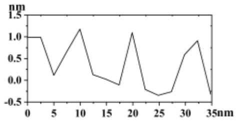</td>
<td>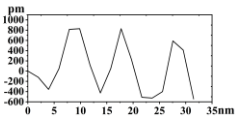</td>
<td>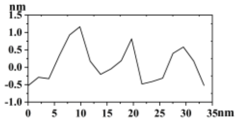</td>
<td>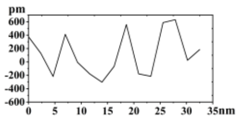</td>
</tr>
<tr><td>86</td><td>87</td><td>88</td><td>89</td><td>90</td></tr>
<tr>
<td>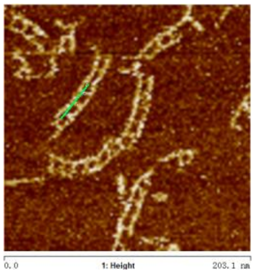</td>
<td>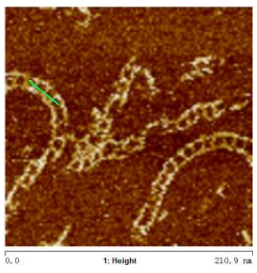</td>
<td>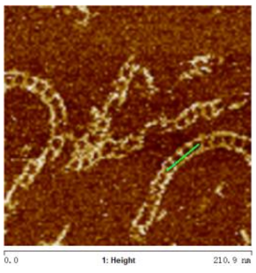</td>
<td>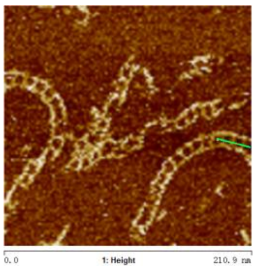</td>
<td>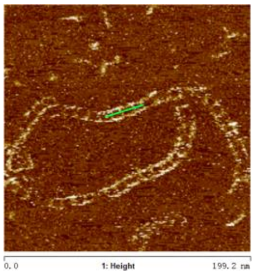</td>
</tr>
<tr>
<td>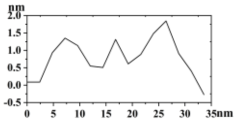</td>
<td>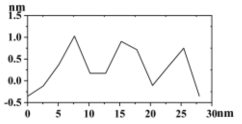</td>
<td>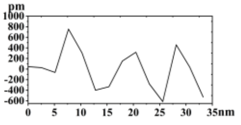</td>
<td>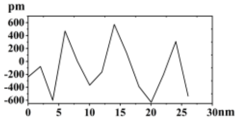</td>
<td>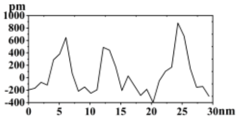</td>
</tr>
</table>

91	92	93	94	95
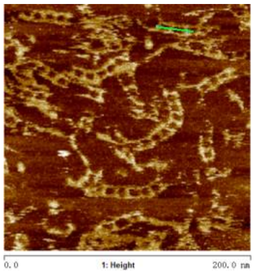	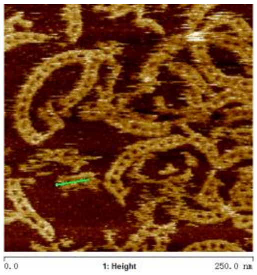	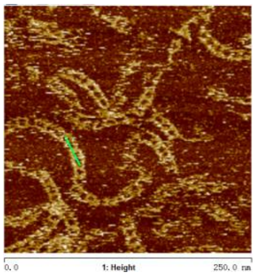	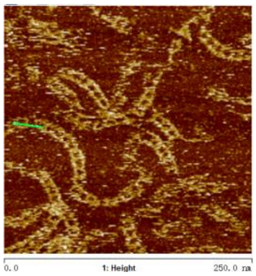	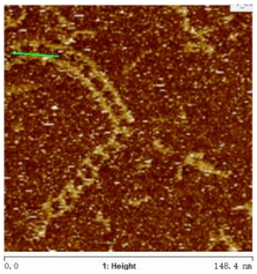
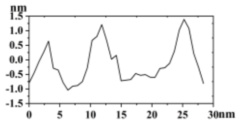	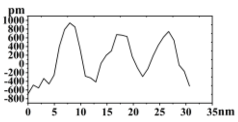	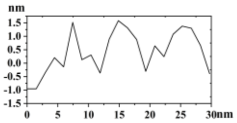	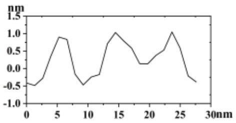	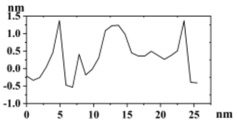
96	97	98	99	100
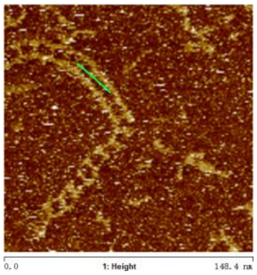	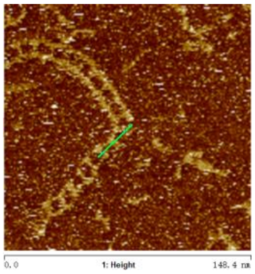	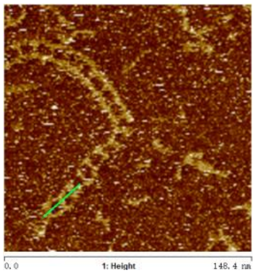	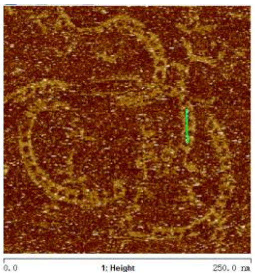	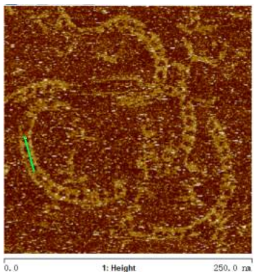
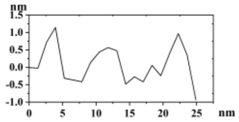	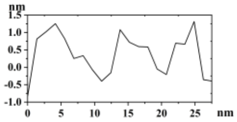	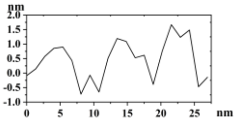	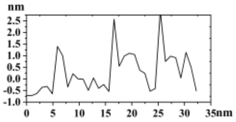	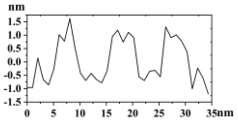
